# Solving the *Myxidium rhodei* (Myxozoa) puzzle: insights into its phylogeny and host specificity in Cypriniformes[Fn FN1]

**DOI:** 10.1051/parasite/2024030

**Published:** 2024-07-01

**Authors:** Dariya Baiko, Martina Lisnerová, Pavla Bartošová-Sojková, Astrid S. Holzer, Petr Blabolil, Michael Schabuss, Ivan Fiala

**Affiliations:** 1 Institute of Parasitology, Biology Centre of the Czech Academy of Sciences České Budějovice 37005 Czech Republic; 2 Faculty of Science, University of South Bohemia in České Budějovice České Budějovice 37005 Czech Republic; 3 Institute for Chemistry and Biology of the Marine Environment (ICBM), Carl von Ossietzky University Oldenburg Oldenburg 26129 Germany; 4 Fish Health Division, University of Veterinary Medicine Vienna 1210 Austria; 5 Institute of Hydrobiology, Biology Centre of the Czech Academy of Sciences České Budějovice 37005 Czech Republic; 6 Profisch OG 1180 Vienna Austria

**Keywords:** Cryptic species, Host specificity, Kidney-infecting *Myxidium* spp., Myxozoa, PCR screening, Phylogeny

## Abstract

*Myxidium rhodei* Léger, 1905 (Cnidaria: Myxozoa) is a kidney-infecting myxosporean that was originally described from the European bitterling *Rhodeus amarus*. Subsequently, it has been documented based on spore morphology in more than 40 other cypriniform species, with the roach *Rutilus rutilus* being the most commonly reported host. This study introduces the first comprehensive data assessment of *M. rhodei*, conducted through morphological, ecological and molecular methods. The morphological and phylogenetic analyses of SSU rDNA sequences of *Myxidium* isolates obtained from European bitterling and roach did not support parasite conspecificity from these fish. In fact, the roach-infecting isolates represent three distinct parasite species. The first two, *M*. *rutili* n. sp. and *M*. *rutilusi* n. sp., are closely related cryptic species clustering with other myxosporeans in the freshwater urinary clade, sharing the same tissue tropism. The third one, *M*. *batuevae* n. sp., previously assigned to *M*. cf. *rhodei*, clustered in the hepatic biliary clade sister to bitterling-infecting *M. rhodei*. Our examination of diverse cypriniform fishes, coupled with molecular and morphological analyses, allowed us to untangle the cryptic species nature of *M. rhodei* and discover the existence of novel species. This underscores the largely undiscovered range of myxozoan diversity and highlights the need to incorporate sequence data in diagnosing novel species.

## Abstract (fr)

*Myxidium rhodei* Léger, 1905 (Cnidaria : Myxozoa) est un Myxosporea infectant les reins qui a été décrit à l’origine chez la bouvière, *Rhodeus amarus*. Par la suite, il a été documenté, sur la base de la morphologie des spores, chez plus de 40 autres espèces de cypriniformes, le gardon *Rutilus rutilus* étant l’hôte le plus fréquemment signalé. Cette étude présente la première évaluation complète des données sur *M. rhodei*, réalisée par des méthodes morphologiques, écologiques et moléculaires. Les analyse morphologiques et phylogénétiques des séquences d’ADNr SSU des isolats de *Myxidium* obtenus à partir de bouvières et de gardons européens n’ont pas confirmé la conspécificité du parasite de ces poissons. En fait, les isolats infectant les gardons représentent trois espèces distinctes de parasites. Les deux premières, *M. rutili* n. sp. et *M. rutilusi* n. sp., sont des espèces cryptiques étroitement apparentées, regroupées avec d’autres Myxosporea du clade urinaire d’eau douce, partageant le même tropisme tissulaire. La troisième, *M. batuevae* n. sp., précédemment attribuée à *M.* cf. *rhodei*, appartient au clade biliaire hépatique, groupe-frère de *M. rhodei* infectant la bouvière. Notre examen de divers poissons cypriniformes, couplé à des analyses moléculaires et morphologiques, nous a permis de démêler la nature cryptique des espèces de *M. rhodei* et de découvrir l’existence de nouvelles espèces. Cela souligne la diversité largement méconnue des Myxozoaires et souligne la nécessité d’incorporer des données de séquence dans le diagnostic de nouvelles espèces.

## Introduction

The subphylum Myxozoa Grassé, 1970 (Cnidaria) is represented by microscopic metazoan endoparasites. Their two-host life cycle involves an invertebrate (bryozoans or annelids) as a definitive host and a vertebrate (mainly fish, amphibians, reptiles, and rarely birds or small mammals) as an intermediate host [18, 42]. Myxozoa encompass more than 2,600 species, making a significant contribution to the biodiversity of cnidarians reaching up to 14,000 described species [43]. Fish, however, remain the focal point of interest in myxozoan research due to the high frequency of records in this host group and their significant importance for the aquaculture sector [40, 44, 52]. Although many myxozoan infections go unnoticed, some, such as those that cause whirling disease and proliferative kidney disease in salmonids, have a substantial impact on wild, free ranging fishes and hatchery fisheries [20, 21].

*Myxidium* Bütschli, 1882 is a polyphyletic myxosporean genus of typically coelozoic (in body and organ cavities), rarely histozoic (in tissues) parasites, encompassing over 230 nominal species [16, 38]. Representatives of this genus can be found in both freshwater and marine environments and are positioned in the oligochaete-infecting (mostly freshwater) and polychaete-infecting (mostly marine) lineage of the myxozoan phylogenetic tree [18, 23]. Morphologically, the genus is characterized by straight, crescent or sigmoid spindle-shaped myxospores [16]. Two pyriform polar capsules are located at the pointed ends of the spore. The sutural line divides the spore into two equal shell valves with a smooth or ridged surface [38]. The spores of *Myxidium* morphologically resemble those of *Zschokkella*, *Ellipsomyxa*, and *Sigmomyxa*, thereby making it challenging to discern the subtle differences between these genera [18].

In total, 23 *Myxidium* species have been described to infect the kidneys of cypriniform fishes in the freshwater ecosystems, with 17 of them having been documented in Eurasia [1, 36, 39]. Molecular data and associated knowledge of phylogenetic relationships for most kidney-infecting *Myxidium* species remain largely unknown [9, 53]. As such, most descriptions are based on morphological data and reports of the host specificity of these parasites [16]. Some species within the investigated genus manifest noteworthy morphological resemblance.

*Myxidium* species infecting the kidneys of cyprinids have been primarily assigned to be *M. rhodei* Léger, 1905*,* a species originally described from the kidney tissue of European bitterling *Rhodeus amarus* more than 100 years ago [36]. Since then, *M*. *rhodei* has been documented from more than 40 freshwater cypriniform species based on morphological evidence, with roach *Rutilus rutilus* considered its most common host [1, 2, 9, 10, 12, 13, 32, 39, 45, 49, 51]. Though the kidney tissue is a target site of spore production, urinary duct, liver, muscles, spleen, heart, swim bladder, and gonads have also been reached by parasite stages in severely infected specimens [3, 4, 8, 9, 13, 14, 31, 49]. Given its broad host range and similar spore morphology in various hosts, *M. rhodei* had been considered a complex of cryptic species and all future *M. rhodei*-like cyprinid isolates were to be assigned as *M.* cf. *rhodei* until molecular data from a type host are available [39]. According to that, two *Myxidium* isolates originating from the roach, were referred to as *M.* cf. *rhodei* and their associated SSU rDNA sequence have represented the first molecular data of *M. rhodei*-like species until now [9]. Nevertheless, sequence data for *M. rhodei* from its type host, the European bitterling, have not been available. Consequently, the phylogenetic position of *M. rhodei* and other kidney-infecting *Myxidium* species has remained unclear.

In this study, we conducted comprehensive screening of cypriniform fish species in European freshwater ecosystems. Our goal was to gather both morphological and sequence data concerning *M. rhodei* and other kidney-infecting *Myxidium* spp. in order to assess their host specificity and explore their phylogenetic relationships. Notably, the inclusion of samples from the type fish host of *M. rhodei* offered compelling evidence to untangle the cryptic species nature of this parasite. The study was a part of a wider project focused on myxozoan diversity in freshwater fish.

## Materials and methods

### Ethics

All fish were euthanized with an overdose of buffered MS-222 (Sigma-Aldrich, St. Louis, MO, USA). Fishing permissions for *R. amarus* were granted by the Nature Conservation Agency of the Czech Republic No. 0016/SOPK/16 and 00720/SOPK/15.

### Sample collection, light microscopy and histology

In total, 583 fish specimens were sampled as part of a larger parasite diversity study, from fish ponds, dams, and rivers across the Czech Republic (511), Bulgaria (44), Belarus (23), and Poland (5) during the period of 2012–2018 (details in Supplementary Table 1).

Obtained fish were dissected in lab conditions and kidneys were examined for the presence of myxozoan infections using the light microscopy (Olympus BX51; Tokyo, Japan) at a magnification of 400× and 1000×. Spores and plasmodia were documented by the light microscope equipped with a digital camera (Olympus DP70; Tokyo, Japan), at 1000× magnification. Spore and polar capsule length and width were derived from digital photographs of 20–40 fresh spores for each *Myxidium* species, following the guidelines of [37] and using ImageJ 1.53e software [50]. Species descriptions of parasites are based on the measurements of spores and polar capsules from single individuals of type fish host species, and when applicable, measurements from additional host species are provided. Measurement values are presented as the average dimension followed by the mean ± standard deviation and maximum and minimum range values of each parameter in parentheses, with all values given in micrometers.

For histological examination, kidney tissues were fixed in Davidson’s fixative for 24 hours, followed by storage in 70% ethanol. The obtained samples were dehydrated in graded alcohol series and routinely embedded in paraffin. Semithin sections were stained using hematoxylin, eosin, and Giemsa (Sigma-Aldrich, St. Louis, MO, USA). If possible, spores were fixed and prepared for scanning electron microscopy (SEM) as described in [33] and examined using a JEOL JEM 1010 field emission scanning electron microscope (JEOL Ltd., Tokyo, Japan).

### DNA isolation, PCR, cloning, sequencing

Small pieces (approx. 5 mm^3^) of all collected kidney tissue samples (*n* = 583) were preserved in 400 μL of TNES urea buffer (10 mM Tris-HCl with pH 8; 125 mM NaCl; 10 mM EDTA; 0.5% SDS and 4 M urea; Sigma-Aldrich, St. Louis, MO, USA). Afterwards, proteinase K (50 lg/mL; Serva, Heidelberg, Germany) was added for an overnight digestion at 55 °C and total DNA was extracted by a standard phenol-chloroform method [24]. The DNA pellet was dissolved in 50–100 μL DNAse-free water and samples were stored overnight at 4 °C before their final storage at −20 °C.

PCR amplification was carried out using Taq Purple polymerase (Top-Bio, Prague, Czech Republic) for most reactions. Moreover, Titanium Taq polymerase (Clontech Laboratories, Mountain View, CA, USA) was used in cases of low product yield when utilizing Taq purple polymerase. Each polymerase was used along with the corresponding manufacturer-provided buffer.

Polymerase chain reactions (PCRs) targeting SSU rDNA were performed in a total volume of 10 μL/reaction, using the polymerase and corresponding buffer and with 250 μM of each dNTPs, 10 pmol of each primer, 1 μL of DNA, and sterile water (final volume of each PCR reaction: 10 μL). To ensure successful amplification of myxozoan SSU rDNA, the samples were initially subjected to amplification using universal eukaryotic primers (ERIB1 + ERIB10 or 18e + 18g; [6, 22]). Subsequently, 1 μL of PCR amplicons from the initial PCR was utilized as a DNA template for a nested PCR with myxozoan-specific (MyxospecF–MyxospecR, MyxGP2F–ACT1R, Myxgen4F–ACT1R; [17, 19, 29, 30]) or species-specific primers (Mrhod511F–Mrhod953R, Mrhodei_sstricF1–Mrhodei_sstricR1; present study) (details in Supplementary Table 2).

The PCR amplification consisted of initial denaturation at 95 °C for 3 min, followed by 30 cycles of a denaturation step at 95 °C for 1 min, an annealing step at 52–64 °C (depending on the primers; Supplementary Table 2) for 30–60 s (in accordance with the expected product length), and an extension step at 72 °C (Taq Purple polymerase) or 68 °C (Titanium polymerase) for 2 min, followed by a final incubation at 72 °C or 68 °C for 10 min (see Supplementary Table 2 for further details regarding the PCR amplification). We aimed to sequence the full length of the SSU rDNA for a type sample. However, smaller fragments of SSU rDNA containing both the conservative as well as variable regions with sufficient number of positions for species determination and its discrimination from closely related species remain informative.

PCR products were extracted by Gel/PCR DNA Fragment Extraction Kit (Geneaid Biotech Ltd., New Taipei City, Taiwan). The chosen PCR products were sent for Sanger sequencing (SEQme, Dobříš, Czech Republic) (at most three positive PCR products from a particular host at specific location). If mixed chromatograms were detected, PCR products were cloned into the pDrive Vector using a PCR Cloning Kit (QIAGEN GmbH, Hilden, Germany). Subsequently, plasmids were transformed into One Shot Top10 Competent *Escherichia coli* cells (Life Technologies, Prague, Czech Republic). In total, 10 colonies were subjected for PCR screen using the M13F and M13R primers in order to identify the colonies carrying an insert. Then, 5 positive colonies were selected for outgrowth and subsequent plasmid purification using the High Pure Plasmid Isolation Kit (Roche Applied Science, Penzberg, Germany), and 3–5 colonies from each PCR product were subjected to Sanger sequencing (SEQme, Dobříš, Czech Republic). Plasmid inserts were Sanger sequenced (SEQme, Dobříš, Czech Republic) in both directions using M13F and M13R primers.

### Alignments, phylogenetic analyses, and proportional distances

A dataset of 91 SSU rDNA sequences (a total length of 1,448 bp) was prepared using MAFFT v7.017 [27] employing the E-INS-i multiple alignment method with gap opening penalty set to 1.23 and gap extension penalty to 0. The entire dataset covered the full diversity of the oligochaete-infecting group of Myxozoa, and representatives from all its subgroups were included. As an outgroup, the representatives of the sister phylogenetic group, the polychaete-infecting group of Myxozoa, were selected, *i.e.*, *Auerbachia pulchra*, *Myxidium gadi*, and *Schulmania aenigmatosa*. The alignment was manually edited and ambiguously aligned regions removed. Maximum likelihood (ML) analysis was performed using RAxML v7.2.8 [52] utilizing the GTR + Γ model of evolution which was selected as the best-fitting model of evolution in jModelTest [46]. Bayesian inference (BI) was carried out using MrBayes v3.0 [48] with the GTR + Γ model. MrBayes was executed to estimate posterior probabilities over 1 million generations *via* 2 independent runs of 4 simultaneous Markov Chain Monte Carlo algorithms with every 100th tree saved. Tracer v1.4.1 [47] was applied to determine the length of the burn-in period. Species-specific genetic divergences were identified from proportional distances of sequences (in %), which were calculated using Geneious Prime v. 2019.0.4 [28] with the dataset previously employed for the phylogenetic analyses.

## Results

### Morphological and molecular identification of kidney-infecting *Myxidium* species

Myxospores and plasmodia of kidney-infecting *Myxidium* spp. were detected in 13% of examined fish specimens (73/583). In detail, developmental stages consistent with the morphological diagnosis of the genus *Myxidium* were observed either through microscopic examination and/or identified molecularly in the kidney tissues of fish species listed in Table 1.

**Table 1:** Data on fish host screened for kidney-infecting *Myxidium* spp. in the present study.

Host fish	Nr of fish	Locality, country abbreviation	GPS coordinates	Prevalence at locality	Prevalence in fish host	Total prevalence
*Alburnus alburnus*	2	Hamerský brook, CZ	49°8′50.7″N; 15°3′56.3″E	50% (1/2)	4% (1/28)	*M. rhodei* (16%; 20/126)
	11	Malše river, CZ	48°53′58.553″N; 14°29′9.738″E	0% (0/11)		
	6	Římov Reservoir, CZ	48°49′58.440″N; 14°29′0.960″E	0% (0/6)		
	9	Švihov Reservoir, CZ	49°40′27.480″N; 15°9′48.600″E	0% (0/9)		
*Barbatula barbatula*	8	Hostačovka brook, CZ	49°48′56.707″N; 15°31′48.786″E	100% (8/8)	73% (8/11)	
	1	Horusický pond, CZ	49°9′22.320″N; 14°40′28.200″E	0% (0/1)		
	2	Zlatý brook, CZ	49°7′46.188″N; 13°34′43.040″E	0% (0/2)		
*Leuciscus leuciscus*	38	Malše river, CZ	48°53′58.553″N; 14°29′9.738″E	13% (5/38)	12% (5/43)	
	2	Jihlava river, Moravská Bránice, CZ	49°4′38.716″N; 16°25′47.257″E	0% (0/2)		
	3	Římov Reservoir, CZ	48°49′58.440″N; 14°29′0.960″E	0% (0/3)		
*Misgurnus fossilis*	1	Lužnice river, CZ	49°3′14.760″N; 14°45′46.800″E	0% (0/1)	20% (2/10)	
	9	Ipuť river, Gomel, BY	52°25′07.3″N; 31°03′50.9″E	22% (2/9)		
*Rhodeus amarus*	3	Bohdanečský pond, CZ	50°5′33.356″N; 15°40′15.460″E	0% (0/3)	12% (4/34)	
	21	Hamerský brook, CZ	49°8′50.7″N; 15°3′56.3″E	9.5% (2/21)		
	5	Jihlava river, Moravská Bránice, CZ	49°4′38.716″N; 16°25′47.257″E	0% (0/5)		
	5	Biebrza river, PL	53°29′13.5″N; 22°40′24.8″E	40% (2/5)		
*Abramis brama*	12	Malše river, CZ	48°53′58.553″N; 14°29′9.738″E	0% (0/12)	4% (2/47)	*M. rutilusi* n. sp.(10%; 20/191)
	3	Obecník pond, CZ	49°48′46.440″N; 15°28′28.920″E	0% (0/3)		
	3	Římov Reservoir, CZ	48°49′58.440″N; 14°29′0.960″E	67% (2/3)		
	29	Švihov Reservoir, CZ	49°40′27.480″N; 15°9′48.600″E	0% (0/29)		
*Leuciscus idus*	1	Lužnice river, CZ	49°3′14.760″N; 14°45′46.800″E	0% (0/1)	47% (6/13)	
	10	Hostačovský pond, CZ	49°50′51.720″N; 15°29′44.160″E	60% (6/10)		
	2	Švihov Reservoir, CZ	49°40′27.480″N; 15°9′48.600″E	0% (0/2)		
*Rutilus rutilus*	7	Hamerský brook, CZ	49°8′50.7″N; 15°3′56.3″E	0% (0/7)	12% (11/94)	
	11	Horusický pond, CZ	49°9′22.320″N; 14°40′28.200″E	0% (0/11)		
	5	Hostačovka brook, CZ	49°48′56.707″N; 15°31′48.786″E	0% (0/5)		
	1	Máchovo lake, CZ	50°35′0.561″N; 14°38′59.413″E	0% (0/1)		
	9	Malše river, CZ	48°53′58.553″N; 14°29′9.738″E	0% (0/9)		
	2	Obecník pond, CZ	49°48′46.440″N; 15°28′28.920″E	100% (2/2)		
	12	Rájský pond, CZ	49°49′45.840″N; 15°28′5.880″E	59% (7/12)		
	3	Římov Reservoir, CZ	48°49′58.440″N; 14°29′0.960″E	66.7% (2/3)		
	5	Velký Tisý pond, CZ	49°3′55.8″N; 14°42′26.5″E	0% (0/5)		
	3	Vožralý pond, CZ	49°0′46.146″N; 15°18′46.707″E	0% (0/3)		
	20	Záblatský pond, CZ	49°6′25.600″N; 14°40′7.551″E	0% (0/20)		
	2	Zlatý brook, CZ	49°7′46.188″N; 13°34′43.040″E	0% (0/2)		
	14	Švihov Reservoir, CZ	49°40′27.480″N; 15°9′48.600″E	0% (0/14)		
*Scardinius erythrophthalmus*	2	Bolevecký rybník	49°46′25.680″N; 13°23′54.240″E	0% (0/4)	3% (1/37)	
	4	Hamerský brook, CZ	49°8′50.7″N; 15°3′56.3″E	0% (0/1)		
	1	Horusický pond, CZ	49°9′22.320″N; 14°40′28.200″E	0% (0/5)		
	5	Obecník pond, CZ	49°48′46.440″N; 15°28′28.920″E	0% (0/2)		
	2	pond in Northern Moravia, CZ	–	12.5% (1/8)		
	8	Rájský pond, CZ	49°49′45.840″N; 15°28′5.880″E	0% (0/4)		
	4	Nežarka river, CZ	49°9′24.480″N; 14°46′5.520″E	0% (0/2)		
	2	Velký Tisý pond, CZ	49°3′55.8″N; 14°42′26.5″E	0% (0/3)		
	3	Záblatský pond, CZ	49°6′25.600″N; 14°40′7.551″E	0% (0/6)		
	6	Švihov Reservoir, CZ	49°40′27.480″N; 15°9′48.600″E	0% (0/6)		
*Alburnus alburnus*	2	Hamerský brook, CZ	49°8′50.7″N; 15°3′56.3″E	0% (0/2)	4% (1/28)	*M. rutili* n. sp.(18%; 43/242)
	17	Malše river, Římov, CZ	48°49′58.440″N; 14°29′0.960″E	6.0% (1/17)		
	9	Švihov Reservoir, CZ	49°40′27.480″N; 15°9′48.600″E	0% (0/9)		
*Squalius cephalus*	35	Malše river, CZ	48°53′58.553″N; 14°29′9.738″E	6% (2/35)	5% (2/39)	
	4	Švihov Reservoir, CZ	49°40′27.480″N; 15°9′48.600″E	0% (0/4)		
*Leuciscus leuciscus*	38	Malše river, CZ	48°53′58.553″N; 14°29′9.738″E	5% (2/38)	5% (2/43)	
	2	Jihlava river, Moravská Bránice, CZ	49°4′38.716″N; 16°25′47.257″E	0% (0/2)		
	3	Římov Reservoir, CZ	48°49′58.440″N; 14°29′0.960″E	0% (0/3)		
*Rutilus rutilus*	7	Hamerský brook, CZ	49°8′50.7″N; 15°3′56.3″E	86% (6/7)	35% (33/94)	
	11	Horusický pond, CZ	49°9′22.320″N; 14°40′28.200″E	0% (0/11)		
	5	Hostačovka brook, CZ	49°48′56.707″N; 15°31′48.786″E	0% (0/5)		
	1	Máchovo lake, CZ	50°35′0.561″N; 14°38′59.413″E	100% (1/1)		
	9	Malše river, Plav, CZ	48°53′58.553″N; 14°29′9.738″E	56% (5/9)		
	2	Obecník pond, CZ	49°48′46.440″N; 15°28′28.920″E	0% (0/2)		
	12	Rájský pond, CZ	49°49′45.840″N; 15°28′5.880″E	0% (0/12)		
	3	Římov Reservoir, CZ	48°49′58.440″N; 14°29′0.960″E	0% (0/3)		
	5	Velký Tisý pond, CZ	49°3′55.8″N; 14°42′26.5″E	100% (5/5)		
	3	Vožralý pond, CZ	49°0′46.146″N; 15°18′46.707″E	0% (0/3)		
	20	Záblatský pond, CZ	49°6′25.600″N; 14°40′7.551″E	80% (16/20)		
	2	Zlatý brook, CZ	49°7′46.188″N; 13°34′43.040″E	0% (0/2)		
	14	Švihov Reservoir, CZ	49°40′27.480″N; 15°9′48.600″E	0% (0/14)		
*Scardinius erythrophthalmus*	2	Bolevecký pond, CZ	49°46′25.680″N; 13°23′54.240″E	0% (0/2)	14% (5/37)	
	4	Hamerský brook, CZ	49°8′50.7″N; 15°3′56.3″E	100% (2/2)		
	1	Horusický pond, CZ	49°9′22.320″N; 14°40′28.200″E	75% (3/4)		
	5	Obecník pond, CZ	49°48′46.440″N; 15°28′28.920″E	0% (0/1)		
	2	pond in Northern Moravia, CZ	–	0% (0/5)		
	8	Rájský pond, CZ	49°49′45.840″N; 15°28′5.880″E	0% (0/2)		
	4	Nežárka river, CZ	–	0% (0/8)		
	2	Velký Tisý pond, CZ	49°3′43.010″N; 14°43′3.661″E	0% (0/4)		
	3	Záblatský pond, CZ	49°6′25.600″N; 14°40′7.551″E	0% (0/2)		
	6	Švihov Reservoir, CZ	49°40′27.480″N; 15°9′48.600″E	0% (0/3)		
	1	Horusický pond, CZ	49°9′22.320″N; 14°40′28.200″E	0% (0/6)		
	2	Zlatý brook, CZ	49°7′46.188″N; 13°34′43.040″E	0% (0/2)		

### Phylogenetic analysis and genetic distances

**Figure 1:**
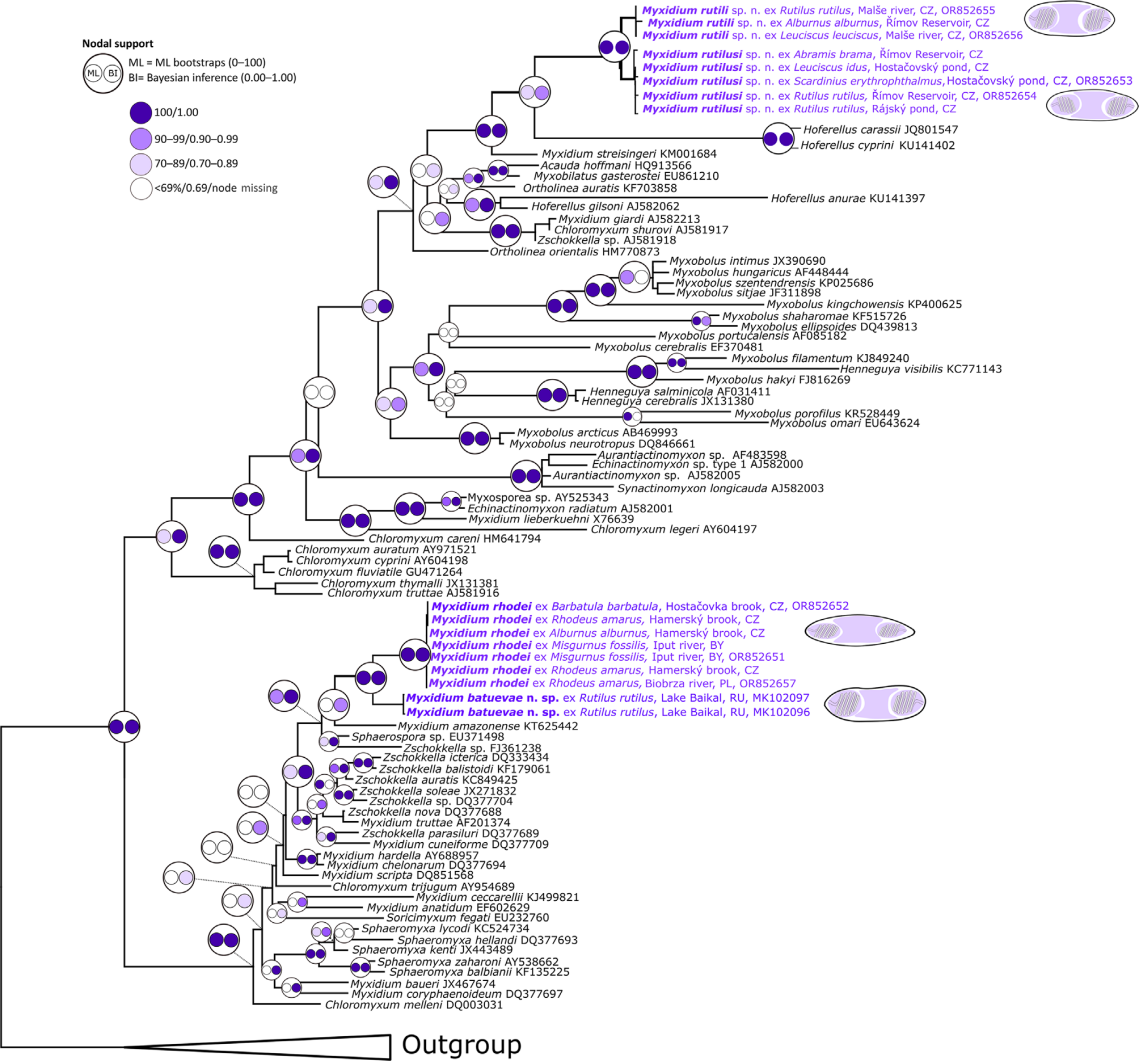
Phylogenetic tree based on SSU rDNA including all sequences of kidney-infecting *Myxidium* spp. and closely related myxozoans. The sequences of *Auerbachia pulchra*, *Myxidium gadi* and *Schulmania aenigmatosa* were used as the outgroup. New identified species are shown in purple and bold. Maximum likelihood/Bayesian inference nodal supports are shown at every node in a circle colored according to the legend on the upper left side.

Partial SSU rDNA sequences of *Myxidium* spp. were obtained from all infected kidney tissues of ten out of 29 examined host species, namely bleak (*n* = 2)*,* chub (*n* = 2), dace (*n* = 5), European bitterling (*n* = 4), freshwater bream (*n* = 2), ide (*n* = 3), roach (*n* = 19)*,* rudd (*n* = 5), stone loach (*n* = 3), and weatherfish (*n* = 2) (at most, three from a particular host at a specific location, *n* = 47). Phylogenetic analyses revealed parasite isolates grouping into two distinct phylogenetic clades of the oligochaete-infecting (freshwater) lineage of myxosporeans (Figure 1). Notably, sequence comparison did not support conspecificity of the isolates from European bitterling and roach-infecting isolates. Indeed, *Myxidium* isolates from roach and other cyprinids clustered apart from the isolates originating from European bitterling and other cyprinids (Figure 1).

Our analysis of newly gathered molecular data facilitated the sequence recognition of “true” *M. rhodei* based on parasite morphology and importantly by sample origin from the type host, the European bitterling. The sequence data of the parasite from its type host (GenBank: OR852657) matched the sequences acquired from the kidney of other cypriniform hosts, *i.e.*, bleak, stone loach (GenBank: OR852652, similarity 99.9% across 632 bp and weatherfish (GenBank: OR852651, similarity 99.9% across 784 bp). Notably, *M. rhodei* fish hosts in this study originated from geographically distant locations in the Czech Republic, Poland, and Belarus. Phylogenetically, *M. rhodei* clustered within the freshwater hepatic biliary clade of myxosporeans represented by a mixture of species of various genera from the biliary and urinary system of fish, amphibians, reptiles, birds, and mammals. In more detail, *M. rhodei* further clustered with the previously published sequences of the roach-infecting *M.* cf. *rhodei* (MK102096–MK102097), and then with gallbladder-infecting *Myxidium amazonense* (GenBank: KT625442), kidney-infecting *Zschokkella* sp. from brown catfish *Ameiurus nebulosus* (GenBank: FJ361238), and *Sphaerospora elwhaiensis* (GenBank: EU371498) (Figure 1).

Other SSU rDNA sequences of *Myxidium* isolates acquired from bleak*,* dace, freshwater bream*,* ide, roach, and rudd clustered apart from “true” *M. rhodei*, within the freshwater urinary clade. Phylogenetic analyses have split these isolates into two closely related lineages (97.9% sequence similarity across 682 bp). Although their morphological traits are hardly discernible (see taxonomic sections below), the level of considerable sequence disparity facilitated describing these closely related isolates as new species. The closest phylogenetic relatives were *Hoferellus cyprini* (GenBank: KU141402), derived from the kidney and urinary bladder of common carp *Cyprinus carpio*, and *H. carassii* (GenBank: JQ801547), found in the kidney and urinary bladder of gibel carp *Carassius auratus*.

### Taxonomic redescription and descriptions

Based on the compiling evidence of morphological, molecular and ecological (host species spectrum, tissue tropism) data, four different species were distinguished. One of them (*M. rhodei*) is redescribed, while the rest are described as new species based on the data obtained in this study (*Myxidium rutili* n. sp. and *Myxidium rutilusi* n. sp.) or in a previous study (*M*. *batuevae* n. sp.; [9]).

### Taxonomic redescription

Phylum Cnidaria Hatschek, 1888

Unranked subphylum Myxozoa Grassé, 1970

Class Myxosporea Bütschli, 1881

Order Bivalvulida Shulman, 1959

Family Myxidiidae Thélohan, 1892

Genus *Myxidium* Thélohan, 1892

### *Myxidium rhodei* Léger, 1905

Type host: *Rhodeus amarus* Bloch, 1782 (Cypriniformes: Cyprinidae), European bitterling.

Other hosts: *Alburnus alburnus* (L.) (Cypriniformes: Cyprinidae), bleak;

     *Barbatula barbatula* (L.) (Cypriniformes: Balitoridae), stone loach;

     *Leuciscus leuciscus* (L.) (Cypriniformes: Cyprinidae), dace;

     *Misgurnus fossilis* (L.) (Cypriniformes: Cobitidae), European weatherfish.

Type locality: France [36].

Other localities: Biebrza river, Poland (53°29′13.5″N; 22°40′24.8″E); Iput river, Belarus (52°25′7.3″N; 31°3′50.9″E); Hamerský brook, Czech Republic (49°8′50.7″N; 15°3′56.3″E); Hostačovka brook, Czech Republic (49°48′56.707″N; 15°31′48.786″E).

Site of tissue development: Coelozoic in kidney glomeruli.

Material deposited: Neotype – DNA and slide with histological section, stored at the Protistological Collection of the Institute of Parasitology, BC CAS, České Budějovice, Czech Republic (IPCAS Pro 79); (European bitterling, type host individual, GenBank: OR852657, 788 bp; stone loach, GenBank: OR852652, 875 bp; European weatherfish, GenBank: OR852651, 861 bp).

Prevalence of infection: Overall prevalence of 16% (20/126), more specifically 12% (4/34) in European bitterling*,* 4% (1/27) in bleak, 73% (8/11) in stone loach, 12% (5/43) in dace and 20% (2/10) in European weatherfish (Table 1).

**Figure 2:**
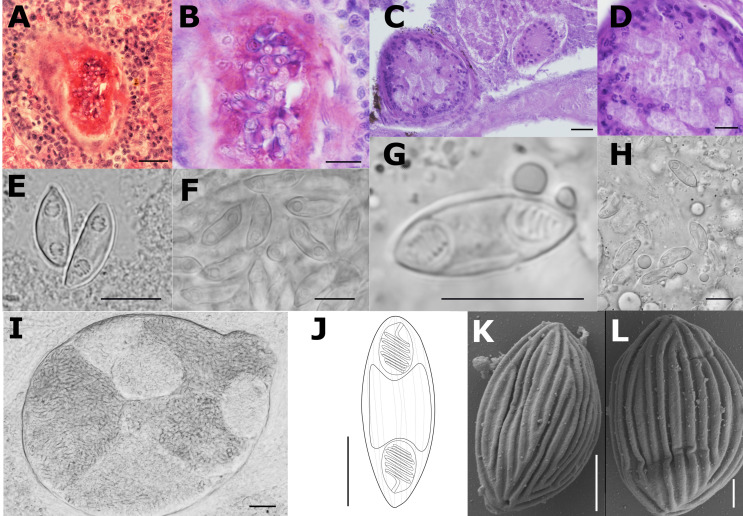
Line drawings, light and scanning electron microscopy (SEM) pictures and histological section of redescribed species *Myxidium rhodei* in this study. A (scale 20 μm), B, E– a cyst in the kidney tissue of *Barbatula barbatula*; C, D – a cyst in the kidney tissue of *Rhodeus amarus*; F – mature spores in the kidney tissue of *B. barbatula*; G, H – mature spores in the kidney tissue of *R. amarus*; I (scale 30 μm) – a cyst filled with spores in kidney tissue of *B. barbatula*; J (scale 5 μm) – line drawing of *M. rhodei* spore, in side view. K, (scale 2.5 μm) L (scale 1.25 μm) – SEM of mature spores. Scale 10 μm unless specified otherwise.

Description of sporogonic stages (type host, single individual): Polysporic plasmodia of oval shape (Figures 2A–2D, 2I).

Description of myxospore (type host, single individual): Mature spore spindle-shaped with pointed poles with 13.7 ± 0.6 (12.5–14.8) μm in length and 5.4 ± 0.4 (4.6–5.9) μm in width; polar capsules pyriform 4.0 ± 0.4 (3.2–4.9) μm in length and 3.1 ± 0.3 (2.6–3.6) μm in width (*n* = 20) (Figures 2G, 2H and 2J); polar filaments with 5 coils per polar capsule (*n* = 20); surface with 9–13 distinctive lines (Figures 2K and 2L).

Description of myxospore (stone loach, single individual): Mature spores elongate tapering at both ends with 14.3 ± 1.0 (12.4–16.7) μm in length and 4.5 ± 0.4 (3.8–5.5) μm in width; polar capsules teardrop-like, sometimes pyriform with 3.0 ± 0.5 (2.1–4.1) μm in length and 2.3 ± 0.19 (1.9–2.8) in width (*n* = 40); polar filaments with 5 coils per polar capsule (*n* = 15); surface with distinctive lines (Figures 2E and 2F).

Pathology: None of the studied fish individuals showed macroscopic pathological changes. Infection intensity was low, with few spore-forming stages observed in all cases (Figures 2A–2D).

Remarks: Léger, in his study [36] reported spores with dimensions of 14.0–15.0 μm in length and 3.8–4.0 μm in width, which were slightly longer and narrower than those observed in European bitterling in the present study. No variation in spore dimensions was observed between hosts. Surface ornamentation, and tissue specificity match the original description of *M. rhodei* [36]. The host spectrum is narrower than previously considered. This parasite species was recognized and molecularly confirmed from four fish species (bleak (*n* = 1), European bitterling (*n* = 4), European weatherfish (*n* = 2), and stone loach (*n* = 3)). Partial SSU rDNA sequence data of *M. rhodei* are provided for the first time in this study. We attempted to obtain the full length SSU rDNA of *M. rhodei*, but failed. Nevertheless, the obtained SSU of *M. rhodei* includes both the conservative as well as variable regions with sufficient number of positions for species determination and its discrimination from closely related species.

### *Myxidium rutili* Baiko et Fiala n. sp

urn:lsid:zoobank.org:act:2B64BAF4-83EE-49FE-8115-C640AAA35356

Type host: *Rutilus rutilus* (L.) (Cypriniformes: Cyprinidae), roach.

Other hosts: *Alburnus alburnus* (L.) (Cypriniformes: Cyprinidae), bleak;

     *Leuciscus leuciscus* (L.) (Cypriniformes: Cyprinidae), dace;

     *Scardinius erythrophthalmus* (L.) (Cypriniformes: Cyprinidae), rudd;

     *Squalius cephalus* (L.) (Cypriniformes: Cyprinidae), chub.

Type locality: Římov Reservoir, Czech Republic (48°49′58.440″N; 14°29′0.960″E).

Other localities: Malše river, Czech Republic (48°53′58.553″N; 14°29′9.738″E); Hamerský brook, Czech Republic (49°8′50.7″N; 15°3′56.3″E); Máchovo lake, Czech Republic (50°35′0.561″N; 14°38′59.413″E).

The site of tissue development: Coelozoic in kidney glomeruli.

Prevalence of infection: Overall prevalence of 18% (43/243), more specifically 35% (33/94) in roach, 4% (1/28) in bleak, 5% (2/43) in dace, 14% (5/37) in rudd and 5% (2/39) in chub (Table 1).

Etymology: Refers to the type host species *Rutilus rutilus*.

Note: The authors of the new taxa are different from the authors of this paper: Article 50.1 and Recommendation 50A of the International Code of Zoological Nomenclature [26].

Material deposited: Hapantotype – DNA and slide with histological section stored at the Protistological Collection of the Institute of Parasitology, BC CAS, České Budějovice, Czech Republic (IPCAS Pro 80); SSU rDNA sequence (roach, type host, single individual, GenBank: OR852655, 896 bp; dace, GenBank: OR852656, 897 bp).

**Figure 3:**
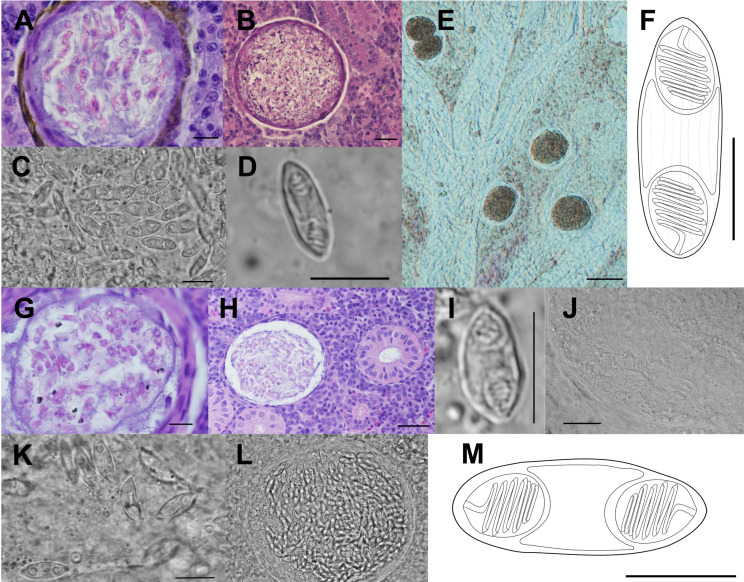
Line drawings, light microscopy pictures and histological section of newly described species in this study. A, B (scale 20 μm), E (scale 100 μm) – a cyst of *Myxidium rutilusi* n. sp. in the kidney tissue of *Rutilus rutilus*; C, D – mature spores of *M. rutilusi* n. sp. in the kidney tissue of *R. rutilus*; F – line drawings of *M. rutilusi* n. sp. spore, in side view. G, H (scale 30 μm), J (scale 30 μm), L – a cyst of *Myxidium rutili* n. sp. in the kidney tissue of *R. rutilus*; I, K – mature spores of *M. rutili* n. sp. in the kidney tissue of *R. rutilus;* M (scale 5 μm) – line drawing of *M. rutili* n. sp. spore, in side view. Scale 10 μm unless specified otherwise.

Description of sporogonic stages: Polysporic plasmodia of round shape (Figures 3G, 3H, 3J and 3L).

Description of myxospore (type host, single individual): Mature spore spindle-shaped, tapering at both ends with 11.6 ± 0.5 (10.6–12.6) μm in length and 4.4 ± 0.5 (3.4–5.2) μm in width (*n* = 20, type host, one individual host); polar capsules 3.7 ± 0.4 (2.9–4.4) μm in length and 2.9 ± 0.3 (2.3–3.4) μm in width (*n* = 20, one individual host); polar filaments with 5 coils per polar capsule (Figures 3I–3M).

Pathology: None of the dissected fish showed pathological changes. Infection intensity with spore-forming stages was low (Figures 3G, 3H, 3J and 3L).

**Figure 4:**
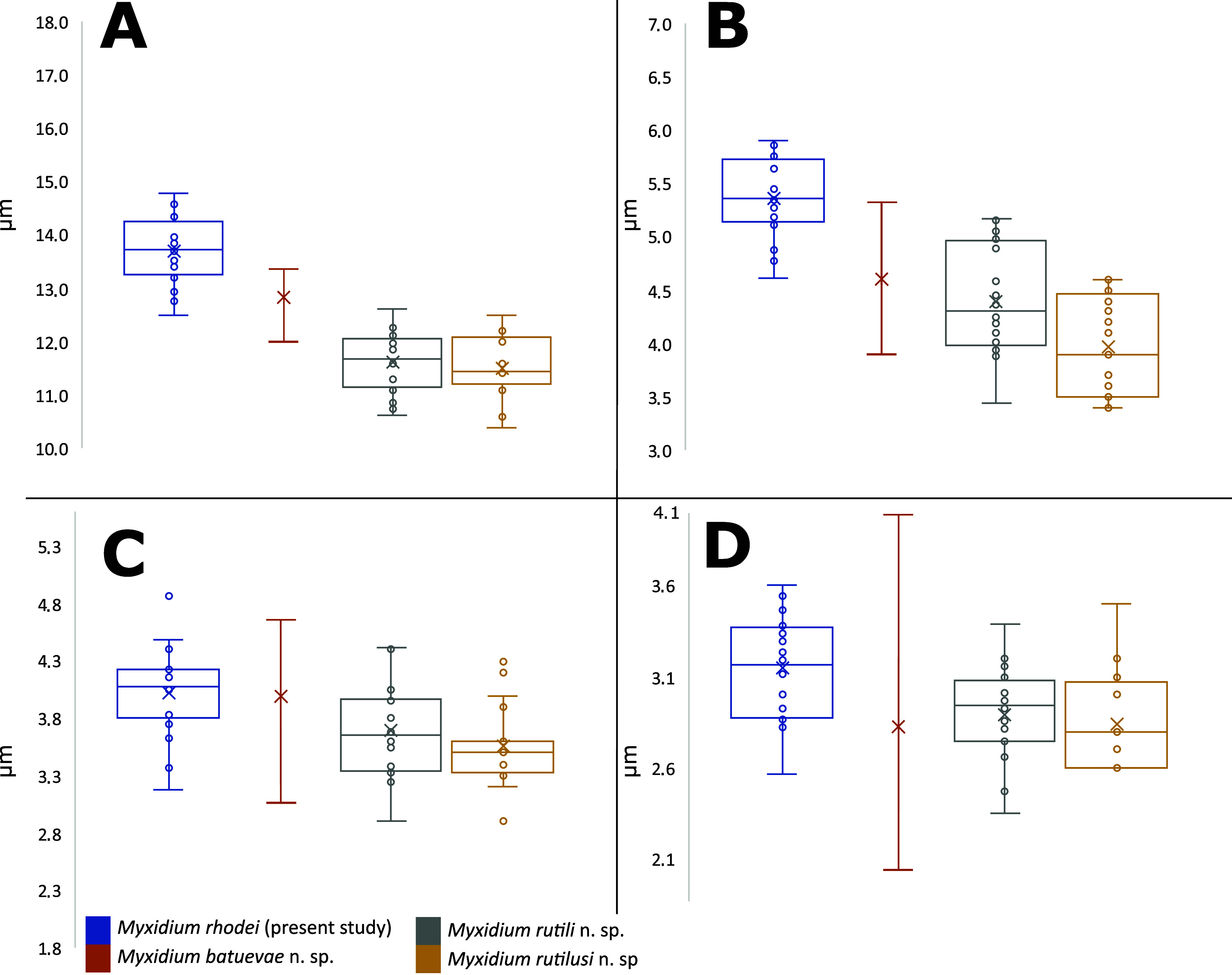
Graphical representation of the measured values (maximum, minimum, average) of spores and polar capsules of selected *Myxidium* spp., color-coded by species according to the legend. A: spore length, B: spore width, C: polar capsule length, D: polar capsule width. The same values of measurements as in descriptions were used.

Remarks: A total of 23 *Myxidium* species have been described in the kidneys of cypriniform hosts. Among these, three species (*M. aletaiense*, *M. macrocapsulatum*, and *M. schulmani*) were described in fish species examined in the present study. Notably, the spores of *M. schulmani* are wider, while spores of *M. aletaiense* are longer than *M. rutili* n. sp. Although *M. rutili* n. sp. and *M. macrocapsulatum* overlap in spore length, width, and host species range, they differ in polar capsule dimensions, spore shape, and polar capsule morphology. Some other species share similar dimensions, but they do not match in the host species spectrum with *M. rutili* n. sp., and there is a lack of morphological or molecular data for direct comparison. Also, *M. rutili* is not strictly host-specific and infects five cyprinid species. The spore morphology closely resembles that of the newly described *M. rutilusi* n. sp; however, the spores are a little wider (Figure 4). Thus, species differentiation primarily relies on disparities in SSU rDNA sequences, with a similarity of 98.0%.

### *Myxidium rutilusi* Lisnerová et Fiala n. sp.

urn:lsid:zoobank.org:act:CCB7A1DA-6437-4316-B25C-34E3B1F0CA12

Type host: *Rutilus rutilus* (L.) (Cypriniformes: Cyprinidae: Leuciscinae), roach.

Other hosts: *Abramis brama* (L.) (Cypriniformes: Cyprinidae: Leuciscinae), bream;

     *Leuciscus idus* (L.) (Cypriniformes: Cyprinidae: Leuciscinae), chub;

     *Scardinius erythrophthalmus* (L.) (Cypriniformes: Cyprinidae: Leuciscinae), rudd.

Type locality: Římov Reservoir, Czech Republic (48°49′58.440″N; 14°29′0.960″E).

Other localities: Rájský pond, Czech Republic (49.8293006N; 15.4683853E); Hostačovský pond, Czech Republic (49°49′45.840″N; 15°28′5.880″E); Obecník pond, Czech Republic (49°48′46.440″N; 15°28′28.920″E).

The site of tissue development: Coelozoic in kidney glomeruli.

Prevalence of infection: Overall prevalence of 11% (20/181), more specifically 12% (11/94) in roach, 4% (2/47) in bream, 3% (1/37) in rudd and 47% (6/13) in ide (Table 1).

Etymology: Refers to the type host species *Rutilus rutilus.*

Note: The authors of the new taxa are different from the authors of this paper: Article 50.1 and Recommendation 50A of the International Code of Zoological Nomenclature [26].

Material deposited: Hapantotype – DNA and slide with histological section stored at the Protistological Collection of the Institute of Parasitology, BC CAS, České Budějovice, Czech Republic (IPCAS Pro 81); SSU rDNA sequences (roach, type host individual, GenBank: OR852654, 908 bp; rudd, GenBank: OR852653, 944 bp).

Description of sporogonic stages (type host, single individual): Polysporic plasmodia of round shape (Figures 3A, 3B and 3E).

Description of myxospore (type host, single individual): Mature spores straight spindle-shaped, tapering at both ends with 11.5 ± 0.6 (10.4–12.5) μm in length and 4.0 ± 0.5 (3.4–4.6) μm in width (*n* = 20); polar capsules 3.6 ± 0.3 (3.2–4.3) μm in length and 2.8 ± 0.3 (2.6–3.5) μm in width; polar filaments with 5 coils per polar capsule; surface with distinctive lines (Figures 3C, 3D and 3F).

Pathology: None of the dissected fish showed macroscopic pathological changes. Infection intensity with spore-forming stages was low (Figures 3A and 3B).

Remarks: A total of three *Myxidium* species have been described to infect the kidney of fish within the subfamily Leuciscinae: *M. aletaiense*; *M. macrocapsulatum*, and *M. schulmani*. However, when considering the shape and measurements of spores, *M. schulmani* and *M. aletaiense* do not align with the characteristics of the newly described *Myxidium* species. Specifically, spores of *M. schulmani* are wider and those of *M. aletaiense* are longer than of *M. rutilusi* n. sp. Although the spore measurements and host range of *M. rutilusi* n. sp. largely overlap with those of *M. macrocapsulatum*, there are large differences in the shape of the myxospores and the morphology of the polar capsules. Nevertheless, there is a lack of available morphological or molecular data for direct comparison. Furthermore, it was observed that *M. rutilusi* n. sp. is not strictly host-specific, but infects Leuciscinae species. Despite the similarities of *M. rutili* n. sp. and spore morphology (Figure 4), species differentiation is primarily based on distinctions in SSU rDNA sequences, with a similarity of 98.0%.

The subsequent description of *M*. *batuevae* n. sp. is based on the data presented in [9]. Our phylogenetic reconstruction revealed that the species tentatively classified as *M.* cf. *rhodei* is, in fact, a distinct species from *M. rhodei*. This finding enabled its formal description as a new species and comparison with other species within the context of our current work.

### *Myxidium batuevae* Lisnerová et Fiala n. sp.

urn:lsid:zoobank.org:act:26E220C0-ED70-48C4-81EC-A68FB8030B3B

Type host: *Rutilus rutilus* (L.) (Cypriniformes: Cyprinidae: Leuciscinae), roach.

Type locality: Chivyrkui Bay (53°46′N; 109°02′E), Lake Baikal, Russia.

The site of tissue development: Histozoic in kidney glomeruli.

Prevalence of infection: 97% (918/942).

Etymology: Refers to the first author of the publication providing data for species description.

Note: The authors of the new taxa are different from the authors of this paper: Article 50.1 and Recommendation 50A of the International Code of Zoological Nomenclature [26].

Materials deposited: SSU rDNA sequences (GenBank: MK102096–MK102097, 1,656 bp and 1,718 bp [9]).

Description of sporogonic stages: Polysporic plasmodia of round shape (Figure 3 in [9]).

Description of myxospore (type host): Mature spores fusiform, tapering at both ends with 12.7 ± 0.1 (11.8–13.4) μm in length and 4.6 ± 0.1 (3.8–5.4) μm in width; polar capsules 4.0 ± 0.1 (3.1–4.7) μm in length and 2.8 ± 0.1 (2.0–4.0) μm in width; polar filaments with 4–5 coils per polar capsule; surface with 18–20 distinct ridges (Figures 1A–1D, Figures 2A and 2B in [9]).

Pathology: Plasmodia caused compression of the glomeruli in the Bowman’s capsules (Figure 3 in [9]).

Remarks: A total of 23 *Myxidium* species have been described in the kidneys of Cypriniform fish. Among these, three species (*M. aletaiense*, *M. macrocapsulatum*, and *M. schulmani*) have been described in fish species examined in the present and previous study [9]. Notably, the spores of *M. aletaiense* and *M. schulmani* are wider, and those spores of *M. macrocapsulatum* are shorter than *M. batuevae* n. sp. Phylogenetically, *M. batuevae* n. sp. is closely related to *M. rhodei* (GenBank: OR852651, similarity 88.7% across 879 bp)*.* The spores of *M. batuevae* n. sp. are shorter than those of *M. rhodei* from its type host*.* As *M. batuevae* n. sp. overlaps with *M. rutili* n. sp. and *M. rutilusi* n. sp. in spore length, width and host species spectrum (Figures 4A–4D), species differentiation primarily relies on disparities in SSU rDNA sequences, with similarities of 70.0% and 68.0% (897 bp, respectively 729 bp), respectively.

### Spore comparison

Spore and polar capsules measurements (*n* = 20) of the four studied species revealed differences in spore and polar capsule dimensions (Figure 4). *Myxidium rhodei* exhibited the greatest spore length (Figure 4A), while *M. batuevae* n. sp., *M. rutili* n. sp. and *M. rutilusi* n. sp. had the shortest spores in terms of length. When it comes to spore width*, M. rhodei* had the widest spores among the species under investigation (Figure 4B). Polar capsules measurements were very similar in all studied cases (Figures 4C and 4D).

## Discussion

Traditionally, prior to the utilization of molecular techniques and the acquisition of phylogenetic data, the taxonomy of the Myxozoa group relied solely on the morphology and morphometry of myxozoan spores and plasmodia [38]. This study presents the first molecular data for *M. rhodei* from the type host as well as for other kidney-infecting myxidiids of similar morphology. Phylogenetic analysis revealed that the isolate of *M. rhodei* originating from the type host European bitterling is distinct from the isolates obtained from roach, a fish species previously reported as a common host of *M. rhodei* [1, 2, 12–14, 32, 39, 45, 49, 51], here newly described as *M. rutili* n. sp. and *M*. *rutilusi* n. sp. These two parasite species from roach and additional fish species constitute two closely related species, whereas *M. rhodei* from European bitterling and additional fish species clusters apart from them in the hepatic biliary clade in a close relation to *M. batuevae* n. sp. from the roach from the Lake Baikal, previously assigned to *M*. cf. *rhodei* ([9]; GenBank: MK102096 and MK102096). The characterization of newly discovered *Myxidium* species is primarily based on spore morphology, host specificity, divergent SSU rDNA gene sequences and phylogenetic analysis [41]. In the case of our newly identified *Myxidium* species (*M. rutili* n. sp. and *M. rutilusi* n. sp.), their differentiation from each other is possible only by their SSU rDNA sequencing, with a distance of approximately 2% (across 683 bp). Differentiation based solely on SSU rDNA, without the possibility to use morphological difference, is a recurring phenomenon in myxozoans, primarily observed in myxobolids characterized by uniform spores with limited morphological features [34, 35]. SSU rDNA analysis represents one of the available methods for distinguishing between species.

### Morphology of spores

Based on the genetic disparity of the European bitterling- and roach-infecting *Myxidium* spp., a thorough revision of spore morphology of *M. rhodei* and its relatives from roach (previously reported as *M. rhodei*) was performed. Our analysis revealed a considerable spore size difference between *M. batuevae* n. sp. from roach and *M. rhodei* from European bitterling with the spores of the latter one being considerably larger.

Since the *M. rhodei* original species description [36], no investigation of this myxosporean has been conducted in its type host European bitterling. Léger, in his study [36] reported spores with dimensions of 14.0–15.0 μm in length and 3.8–4.0 μm in width, which were slightly longer and narrower than those observed in European bitterling in the present study. In comparison, spore dimensions of *Myxidium* from roach kidneys reported by [13] were 10.0–15.0 μm in length by 4.6–5.4 μm in width. Even a smaller spore size was reported by [3], *i.e.*, 10.0 ± 0.9 (9.0–12.0) μm in length by 4.0 ± 0.8 (3.0–5.0) μm in width (British fish isolates) and 9.7 ± 1.1 (9.0–13.0) μm in length by 3.6 ± 0.7 (3.0–5.0) μm in width (Greek fish isolates). Reports of *M*. cf. *rhodei* from chub and Iberian nase *Pseudochondrostoma polylepis* by [1, 49] provided dimensions similar to those of [3, 13]. Our measurements of roach-infecting *Myxidium* spp. fell within the range reported in the literature. Moreover, spore measurements and comparisons of *M. rutili* n. sp. and *M. rutilusi* n. sp. were performed, and no differences in spore dimensions were found between two studied species (Table 2, Figure 4), which corresponds to their close phylogenetic relationship.

**Table 2:** Comparison of newly described species with kidney-infecting *Myxidium* spp.

Species	Tissue	Host	Spore	Polar capsules	GenBank accession	References
*M. aletaiense* Zhao et Ma, 1994	Kidney	*Leuciscus idus*	13.0 (12.8−13.2) × 5.0 (4.9−5.2)	4.1 (4.0−4.3) × 2.1 (1.8−2.5)	*NA*	[15]
*M. cirrhinae* Chen et Hsieh, 1984	Kidney	*Cirrhinus molitorella*	13.9 (12.2−15.3) × 5.8 (5.3−6.8)	3.7 (3.4−3.9) × 3.6 (3.1−4.1)	*NA*	[15]
*M. barbatulae* Cepedé, 1906	Kidney	*Barbatula barbatula*	12−15 × 4−5	5 × 2.5−3	*NA*	[39]
*M. chongqingense* Ma, 1992	Kidney	*Cyprinus carpio*	15.2 (16.2−17.6) × 6.5 (5.6−7.4)	5.5 (5.0−6.0) × 3.2 (2.5−3.5)	*NA*	[15]
*M. ctenopharyngodonis* Akhmerov, 1960	Kidney	*Ctenopharyngodon idella*	18.0−23 × 5−6.5	3.4−5.8 × 3.3−4.0	*NA*	[15]
*M. cyprini* Akhmerov, 1960	Kidney	*Cyprinus carpio haematopterus*	12−13 × 4−5	3×3	*NA*	[15]
*M. histophilum* Thélohan, 1894	Kidney, ovary	*Phoxinus phoxinus*	15	*NA*	*NA*	[15]
*M. hupehense* Chen et Hsieh, 1984	Kidney, Gallbladder	*Carassius auratus auratus*	14.3 (13.2−15.6) × 4.9 (4.8−5.4)	3.7 (3.6−4.2) × 2.9 (2.6−3.0)	*NA*	[15]
		*Semilabeo prochilus*			*NA*	[15]
		*Gnathopogon argentatus*			*NA*	[15]
*M. macrocapsulatum* Schuurmans et Stekhoven, 1920	Kidney	*Scardinius erythrophthalmus*	9.8−11.6 × 2.8−5	2.8−4.5 × 2.8−4.5	*NA*	[15]
*M. mendehi* Fomena et Bouix, 1994	Kidney	*Barbus guirali, B. martorelli*	9.9 (7.8−13.2) × 4.1 (3.1−4.9)	3.4 (2.7−4.5) × 2.3 (1.8−3.1)	*NA*	[15]
*M. misgurni* Chen et Hsieh, 1984	Kidney	*Misgurnus anguillicaudatus*	12.1 (10.2−13.9) × 5.1 (4.3−5.6)	2.8 (2.2−3.6) × 2.9 (2.6−3.4)	*NA*	[15]
*M. ochengense* Chen et Hsieh, 1984	Gallbladder, Kidney, urinary bladder	*Carassius auratus auratus*	9.8 (9.4−10.8) × 5.2 (4.8−5.4)	3.4 (3−3.6) × 3.1 (3.0−3.2)	*NA*	[15]
*M. osteochili* Chen et Hsieh, 1984	Kidney	*Osteochilus salsburyi*	11.8 × 4.4	3.0 × 2.4	*NA*	[15]
*M. procyprisi* Ma et Zhao, 1996	Kidney	*Procypris rabaudi*	12.6 (12.2−12.8) × 4.9 (4.8−5.2)	3.7 (3.2−4.0) × 2.2 (1.6−2.4)	*NA*	[15]
*M. pseudogobii* Akhmerov, 1960	Kidney	*Pseudogobio rivularis*	13−14 × 4.5−5.0	4.4 × 2.8−3	*NA*	[15]
*M. rhinogobie* Ma, 1993	Kidney	*Rhinogobio ventralis*	12.0 (11.3−12.1) × 3.3 (3.2−3.7)	3.3 (3.0−3.7) × 2.0 (1.5−2.2)	*NA*	[15]
*M. rhodei* Leger, 1905	Kidney	*Alburnus alburnus, Barbatula barbatula, Leuciscus leuciscus,Misgurnus fossilis, Rhodeus amarus*	14−15 × 3.8−4	4.5 × 4.5	*NA*	[36]
			13.7 (12.5−14.8) × 4.0 (4.6−5.9)	4.0 (3.2−4.9) × 3.1 (2.6−3.6)	OR852651, OR852652, OR852657	Present study
*M. batuevae* n. sp.	Kidney	*Rutilus rutilus*	12.7 (11.8−13.4) × 4.6 (3.8−5.4)	4.0 (3.1−4.7) × 2.8(2.0−4.0)	MK102096, MK102097	[9]
*M.* cf. *rhodei*	Kidney	*Rutilus rutilus*	10−15 × 4.6−5.4	3.6−4.4 × 2.8−3.6	*NA*	[12]
	Kidney	*Rutilus rutilus*	12.5 (11.4−13.4) × 5.2 (4.5−5.2)	4.0 (3.5−5.0) × 3.2 (2.5−4.0)	*NA*	[31]
	Muscle	*Rutilus rutilus*	12.8 (11.9−13.9) × 4.2 (4.0−5.0)	4.3 (3.5−5.0) × 3.0 (2.8−3.5)	*NA*	[31]
	Kidney	*Squalius cephalus, Chondrostoma polylepis*	12.1 (10.5−15.0) × 5.1 (4−6)	3.4 (2.8−4.5) × 2.5 (2.0−3.5)	*NA*	[2]
	Kidney, muscle, liver	*Rutilus rutilus*	12.1 × 4.6	3.9 × 3.0	*NA*	[31]
	Kidney	*Rutilus rutilus*	10.0 (9.0−12.0) × 4.0 (3.0−5.0)	3.7 (3.0−4.0) × 3.5 (3.0−4.0)	*NA*	[3]
	Kidney	*Rutilus rutilus*	9.7 (9.0−13.0) × 3.6 (3.0−5.0)	3.4 (3.0−4.0) × 3.8 (3.0−4.0)	*NA*	[3]
	Gallbladder, kidney, gonads	*Carassius auratus, Leptobotia elongata*	15.6 (13.9−17.4) × 4.7 (3.5−5.8)	4.4 (3.5−5.2) × 3.4 (2.9−3.5)	*NA*	[11]
	Kidney	*Abramis brama, Squalius cephalus, L. leuciscus, Phoxinus phoxinus, R. rutilus*	11.8 (10.9−12.8) × 4.3 (3.7−4.7)	3.4 (2.8−3.8) × 2.4 (1.8−3.1)	*NA*	[39]
*M. schulmani* Chernova, 1970	Kidney	*Rutilus rutilus*	13.3−14.0 × 7.3	4.0−4.6 × 3.3−4.0	*NA*	[15]
*M. shamama* Ali, Sakran et AbdelBaki, 1999	Kidney	*Labeo niloticus*	15.9 (14.8−16.8) × 6.6 (5.6−7.2)	4.1 (3.84.4) × 3.7 (3.6−4.0)	*NA*	[15]
*M. spinosum* Li et Nie, 1973	Kidney, urinary bladder	*Aristichys nobilis*	8.0 (6.4−8.4) × 9.0 (7.2−10.8)	3.1 (2.4−3.6) × 3.0 (2.4−3.6)	*NA*	[15]
		*Hypophthalmichthys molitrix*			*NA*	[15]
		*Gnathopogon argentatus*			*NA*	[15]
*M. tictoi* Fariyam Kaur et Abidi, 2020	Kidney	*Puntis ticto*	13.1 (11.5−14.2) × 4.7 (4.1−5.4)	3.3 (2.5−3.9) × 2.4 (1.9−2.9)	*–*	[16]
*M. yibinense* Zhao et Ma, 1995	Gills, gallbladder, kidney, liver, mesenteries, testes	*Garra pingi pingi*	11.4 (11.0−12.0) × 7.7 (7.2−8.0)	4 × 3.2	*NA*	[15]
*M. streisingeri* Whipps, Murray et Kent, 2015	Kidney	*Danio rerio*	8.3 (7.4−9.3) × 5.2 (4.5−5.6)	3.0 (2.5−3.5) × 3.0(2.5−3.5)	KM001684	[53]
*M. rutili* n. sp.	Kidney	*Alburnus alburnus, Squalius cephalus, L. leuciscus, Rutilus rutilus, Scardinius erythrophthalmus*	11.6 (10.6−12.6) × 4.4 (3.4−5.2)	3.7 (2.9−4.4) × 2.9 (2.3−3.4)	OR852655, OR852656	Present study
*M. rutilusi* n. sp.	Kidney	*Abramis brama, Leuciscus idus, Rutilus rutilus, Scardinius erythrophthalmus*	11.5 (10.4−12.5) × 4.0 (3.4−4.6)	3.6 (3.2−4.3) × 2.8 (2.6−3.5)	OR852653, OR852654	Present study

### Host specificity

Since its first record more than 100 years ago [36], *M. rhodei* was reported from more than 40 cyprinid species; however, findings were based only on morphological data [1, 2, 10, 13, 14, 32, 39, 49, 51]. Based on our study, *M. rhodei* infects different hosts than *M. rutili* n. sp. and *M. rutilusi* n. sp. Even when these three species were recognized at a shared locality (Hamerský brook, Czech Republic), they were found in different host species. Analogically, ide and bleak hosted all of these abovementioned myxosporeans; however, the fish specimens never originated from the same locality. As such, the three studied species were not found in co-infections in any host and locality. Both closely related *M. rutili* n. sp. and *M. rutilusi* n. sp. overlapped in infecting roach and rudd, however at different localities, while other myxidiids were not identified in these hosts in our study. *Myxidium rutili* n. sp. and *M. rutilusi* n. sp. both co-occurred at a single locality (Římov Reservoir, Czech Republic); however, in different fish species. No fish specimens were co-infected by *M. rutili* n. sp. and *M. rutilusi* n. sp. A certain degree of within-host-species competition might act as a factor for spatiotemporal and spatial separation of the infections during the intrapiscine parasite development in these hosts, as reported previously for other myxozoans [7, 25, 32].

*Myxidium batuevae* n. sp. was recognized to be strictly host specific given by its report from a single host in Lake Baikal (as documented by [9]). However, a previous study [9] did not encompass the examination of other cyprinid fish, leaving the question of the host range of this species unresolved. In contrast, the rest of the studied *Myxidium* species are generalists infecting a relatively wide host range of cypriniform fishes. These fish species are considered to be their target fish hosts, as sporogony was observed.

### Phylogenetic position of *Myxidium* spp.

Following the general trend of myxozoan phylogenetic grouping according to tissue tropism [17], the kidney-infecting *M. rutili* n. sp. and *M. rutilusi* n. sp. clustered with other myxosporeans from the excretory system. On the other hand, the kidney-infecting *M. rhodei* and *M*. *batuevae* n. sp. clustered unexpectedly within the hepatic biliary clade. Based on the tree topology, we assume that the common ancestor of this clade was probably a *Myxidium/Zschokkella*-type species infecting the biliary system of fish and with some species turning to be generalists in regard to tissue tropism range whose descendants might have switched to the exclusive exploitation of the urinary system. Interestingly, these *Myxidium* species have analogously developed a spore morphology that closely resembles that of *M*. *rutili* n. sp. and *M*. *rutilusi* n. sp., which belong to a myxosporean evolutionary lineage that is relatively distant. The spore morphology appears to be influenced by the location of infection within the fish host. When spores develop within large plasmodia situated in specific tissues (such as the kidney in this case), they tend to adopt the most efficient shape to fill the available space, a phenomenon well-documented in various *Henneguya* species [15]. The elongated, spindle-shaped *Myxidium* spore with tapered ends represents an optimal form to fit the limited space within the plasmodium, constrained by the surrounding tissue pressure of the glomerular capsule. This might explain an evolutionary pressure resulting in homoplastic myxosporean spore morphology.

The nearly identical morphology and dimensions of myxospores were also observed for *M. rhodei* and *Myxidium pfeifferi* Auerbach, 1908, a gallbladder myxozoan parasite of cyprinids (mainly roach) originally described from tench *Tinca tinca* and commonly reported from roach [3, 5]. Spores of *M*. *pfeifferi* are primarily observed in the gallbladder, yet they develop within plasmodia located in the bile ducts. Unfortunately, the absence of molecular data for *M. pfeifferi* prevents untangling this puzzle. The phylogenetic position of *M. rhodei* and *M. batuevae* n. sp. within the freshwater hepatic biliary clade leads to an assumption that *M. pfeifferi* may be closely related to these kidney-infecting species and could potentially serve as a link between the gallbladder- and kidney-infecting species within this clade. Unfortunately, the absence of molecular data for *M. pfeifferi* prevents us from performing genetic comparisons.

While most samples originated from the Czech Republic, the inclusion of specimens from other locations (Poland, Belarus, and Bulgaria) provided valuable insights into the geographical distribution of *M. rhodei*. No infection with *M. rhodei* was molecularly recorded in the twelve examined *R. amarus* from Bulgaria. We consider that this was likely due to the limited number of tested fish. Furthermore, sequences obtained from European bitterling*,* bleak, stone loach and weatherfish were nearly identical, despite these fish being collected from three geographically distant regions (Poland and the Czech Republic for European bitterling, the Czech Republic for bleak and stone loach, and Belarus for weatherfish). Although no parasite data were obtained from the type locality of *M. rhodei* (France), the interconnection of waterways and lack of reproductive isolation among western and central European populations of bitterling [11], as well as the absence of genetic differences in *M. rhodei* between Polish and Czech bitterlings, strongly suggest the conspecificity of *M. rhodei* across French and other European habitats. Based on these data, we infer that *M. rhodei* infects a range of hosts in Europe and possibly parts of West Asia, and exhibits pronounced morphological and molecular similarity across its distribution.

## Conclusion

The present study aimed at determining the phylogenetic position of kidney-infecting *Myxidium* spp. in Cypriniformes based on SSU rDNA sequences. The phylogenetic analyses successfully resolved the positioning of *M. rhodei*. Additionally, these analyses revealed that *M. rhodei*, previously reported from roach or freshwater bream [1, 2, 9, 10, 12–14, 32, 39, 45, 49, 51], had been misidentified and does not infect these cyprinids. Instead, three different *Myxidium* species with nearly identical morphological characteristics but well-separated phylogenetic positions, can be found in roach. Comprehensive species descriptions are provided within this study. Notably, *M. batuevae* n. sp. displayed a distinct pattern of strict host specificity, while *M. rutili* sp. n., *M. rutilusi* sp. n., and *M. rhodei* exhibited a broader host range, although not as extensive as initially considered.

To sum up, our thorough examination of diverse cypriniform fishes, followed by molecular and morphological analyses of observed myxidiids, enabled us to untangle the cryptic species nature of *M. rhodei* and discover additional novel cryptic species. This underscores that the full extent of myxozoan diversity remains largely undiscovered and highlights the need to incorporate sequence data in the diagnosis of new myxozoan species.

## Supplementary material

The supplementary material of this article is available at https://www.parasite-journal.org/10.1051/parasite/2024030/olm. *Supplementary Table 1*: Fish species and their numbers examined in present study. *Supplementary Table 2*: Primers used in the present study.
