# Supplementary material for: Solving the Myxidium rhodei (Myxozoa) puzzle: insights into its phylogeny and host specificity in Cypriniformes
Source: Parasite. 2024 Jul 1;31:35. doi: 10.1051/parasite/2024030 (PMC11216160; doi:10.1051/parasite/2024030)
Supplement: Supplementary file 1 — Supplementary Table 1: Fish species and their numbers examined in present study. [file parasite-31-35-s1.pdf]

Supplementary Table 1. Fish species and their numbers examined in present study.

Host	Family: Subfamily	Number of individuals
Abramis brama	Cyprinidae: Leuciscinae	47
Alburnoides bipunctatus	Cyprinidae: Alburninae	10
Alburnus alburnus	Cyprinidae: Alburninae	28
Aspius aspius	Cyprinidae: Leuciscinae	9
Barbatula brabatula	Balitoridae	11
Barbus barbus	Cyprinidae: Barbinae	8
Blicca bjorkna	Cyprinidae: Leuciscinae	12
Carassius auratus	Cyprinidae: Cyprininae	11
Carassius gibelio	Cyprinidae: Cyprininae	1
Carassius carassius	Cyprinidae: Cyprininae	20
Cobitis elongatoides	Cobitidae	10
Ctenopharyngodon idella	Cyprinidae: Squaliobarbinae	6
Cyprinus carpio	Cyprinidae: Cyprininae	16
Gobio gobio	Cyprinidae: Gobioninae	31
Hypophthalmichthys nobilis	Cyprinidae: Xenocyprinae	2
Chondrostoma nasus	Cyprinidae: Leuciscinae	2
Leuciscus idus	Cyprinidae: Leuciscinae	13
Leuciscus leuciscus	Cyprinidae: Leuciscinae	43
Misgurnus fossilis	Cobitidae	10
Phoxinus phoxinus	Cyprinidae: Leuciscinae	16
Pseudorasbora parva	Cyprinidae: Gobioninae	10
Rhodeus amarus	Cyprinidae: Acheilognathinae	34
Rutilus rutilus	Cyprinidae: Leuciscinae	94
Salmo trutta	Salmoniformes: Salmonidae	15
Scardinius erythrophthalmus	Cyprinidae: Leuciscinae	37
Sabanejewia bulgarica	Cobitidae	5
Squalius cephalus	Cyprinidae: Leuciscinae	39
Tinca tinca	Cyprinidae: Tincinae	36
Vimba vimba	Cyprinidae: Leuciscinae	7
**Total**		**583**
