# Supplementary material for: Solving the Myxidium rhodei (Myxozoa) puzzle: insights into its phylogeny and host specificity in Cypriniformes
Source: Parasite. 2024 Jul 1;31:35. doi: 10.1051/parasite/2024030 (PMC11216160; doi:10.1051/parasite/2024030)
Supplement: Supplementary file 2 — Supplementary Table 2: Primers used in the present study. [file parasite-31-35-s2.pdf]

Supplementary Table 2 Primers used in present study.

**Primer name**	**Sequence (5'→3')**	**Annealing temperature (˚C)**	**References**
ERIB1	ACCTGGTTGATCCTGCCAG	60	[6]
ERIB10	CTTCCGCAGGGTTCACCTACGG		
18e	TGGTTGATCCTGCCAGT	64	[22]
18g	GGTAGTAGCGACGGGCGGTGTG		
MyxospecF	TTCTGCCCTATCAACTTGTTG	54	[17]
MyxospecR	GGTTTCNCDGRGGGMCCAAC		
MyxGP2F	WTGGATAACCGTGGGAAA	58	[29]
Act1R	AATTTCACCTCTCGCTGCCA		[19]
Myxgen4F	GTGCCTTGAATAAATCAGAG	58	[28]
Act1R	AATTTCACCTCTCGCTGCCA		[19]
Mrhod511F	GTTTCCTATATGGATAATCATACTGG	52	**Present study**
Mrhod953R	CATCTCATAAGACATAATGGTCAAC		
Mrhodei_sstric_F1	AAGGATTCGACACTGGAATG	60	**Present study**
Mrhodei_sstric_R1	TGCGGGTATATACATAGCGCC		
